# Supporting mental health of medical students: Needs and demands concerning an e-mental health application within medical education 

**DOI:** 10.3205/zma001765

**Published:** 2025-06-16

**Authors:** Catharina Grüneberg, Alexander Bäuerle, Sophia Karanukaran, Dogus Darici, Christoph Jansen, Nora Dörrie, Sven Benson, Martin Teufel, Anita Robitzsch

**Affiliations:** 1 University of Duisburg-Essen, LVR-University Hospital, Clinic for Psychosomatic Medicine and Psychotherapy, Essen, Germany; 2 University of Duisburg-Essen, Center for Translational Neuro- and Behavioral Sciences (C-TNBS), Essen, Germany; 3 University of Münster, Institute of Anatomy and Neurobiology, Münster, Germany; 4 University of Duisburg-Essen, University Hospital Essen, Institute for Medical Education, Essen, Germany

**Keywords:** mHealth, medical education, medical students, user-centered design

## Abstract

**Background:**

Digital technologies are increasingly integrated into medical education and healthcare services. Studies have demonstrated the efficacy of e-mental health approaches, offering cost-effective, user-friendly, anonymous, and geographically flexible support.

**Objective:**

This study aims to investigate the needs and demands of medical students regarding the design of an e-mental health application within the context of medical education to foster stress management and personal skills.

**Methods:**

A cross-sectional study was conducted from November 2022 to July 2023 at the University of Duisburg-Essen, Germany. *N*=229 students were incorporated in the final data evaluation. The survey comprised standardized and established self-generated inquiries. Needs and demands were analyzed descriptively. A cluster analysis was conducted to explore hypothetical subgroups. Differences and similarities of the clusters were compared.

**Results:**

Most students expressed a preference for smartphone or tablet accessibility, with sessions lasting between 10 to 20 minutes during stressful situations and on an as-needed basis. Participants indicated a desire for information and practical exercises related to self- and time-management, self-esteem and confidence, coping with helplessness, learning methodologies, and self-care/resilience. Video, downloadable, and audio content along with access to expert guidance, were deemed valuable. *K*-medoids clustering revealed a low and high burden cluster.

**Conclusions:**

By aligning with specific needs and demands of target populations, e-mental health apps with enhanced usability and a more user-focused approach can be developed to establish a blueprint for an e-mental health app tailored to their requirements.

## Zusammenfassung

**Hintergrund:**

Digitale Technologien werden zunehmend in die medizinische Ausbildung sowie gesundheitliche Versorgung integriert. Die Wirksamkeit von E-Mental-Health-Angeboten ist wissenschaftlich belegt und ermöglichen kosteneffiziente, benutzerfreundliche, anonyme und ortsunabhängige Unterstützung.

**Zielsetzung:**

Ziel der vorliegenden Studie ist die Erhebung der Bedürfnisse von Medizinstudierenden im Hinblick auf die Gestaltung und Anforderungen an eine E-Mental-Health-Anwendung zur Förderung von Stressbewältigung und persönlicher Kompetenzen im Rahmen der medizinischen Ausbildung.

**Methodik:**

Im Zeitraum von November 2022 bis Juli 2023 wurde eine Querschnittsstudie an der Universität Duisburg-Essen durchgeführt. In die finale Datenanalyse gingen *N*=229 Studierende ein. Die Studie umfasste standardisierte sowie eigens entwickelte Erhebungsinstrumente. Bedürfnisse und Anforderungen wurden deskriptiv ausgewertet. Eine Clusteranalyse untersuchte potenzielle Subgruppen. Unterschiede und Gemeinsamkeiten der Gruppen wurden verglichen.

**Ergebnisse:**

Die Studierenden bevorzugten die Nutzung via Smartphone oder Tablet mit Einheiten von 10 bis 20 Minuten Dauer, in akuten Stresssituationen und bei Bedarf. Inhalte zu Selbst-/Zeitmanagement, Selbstwertgefühl/-sicherheit, Umgang mit eigener Hilflosigkeit, Lernmethoden sowie Selbstfürsorge/Resilienz wurden als besonders wichtig eingestuft. Formate wie Video-, Audio- und Downloadmaterialien sowie Kontakt zu Expert:innen wurden als hilfreich eingeschätzt. Die *k*-Medoids-Clusteranalyse ergab eine Gruppe mit erniedrigter und eine mit erhöhter mentaler Belastung.

**Schlussfolgerung:**

Die Entwicklung nutzerzentrierte und bedürfnisorientierter E-Mental-Health-Apps mit Ausrichtung auf spezifische Bedürfnisse und Anforderungen der Zielgruppe kann als Grundlage für eine bedürfnisorientierte E-Mental-Health App für Medizinstudierende dienen.

## 1. Introduction

### 1.1. Background

Digital technologies are increasingly integrated into medical education and healthcare services, offering cost-effective, user-friendly, anonymous, and geographically flexible support [1], [2], [3]. Participatory designs prioritizing the future users’ needs and demands during the development of digital health apps are paramount for optimizing usability and acceptance [4], [5].

The necessity for preventive initiatives targeting university students, with a specific emphasis on stress management, was highlighted in a 2023 health report from a German health insurance provider describing that two-thirds of German students are burdened by stress-related health issues [6]. Medical students are susceptible to heightened stress levels and associated conditions, including anxiety, depression, burnout, and suicidal ideation [7], [8], [9], [10], [11], [12]. The challenge of fostering self-care practices and mental health awareness due to low rates of help-seeking behavior and barriers such as mental health stigma among medical students and physicians is well-documented [13], [14], [15], [16], [17], [18], [19], [20], [21]. Consequently, supportive programs that enhance mental well-being among medical students are needed [22]. 

Participatory design approaches present promising avenues for developing accessible solutions tailored to the preferences of the target demographic, with the potential to mitigate barriers and enhance user satisfaction [4], [23], [24]. By focusing on a user-centered design approach, the quality and usage of e-health apps (electronic health applications) may be improved, and high-drop-out rates reduced [25], [26]. The imperative for implementing interventions targeting university students, particularly medical students, is evident [16], [27], [28]. A study in 2021 described positive views among medical students regarding evidence-based Internet- and mobile-based interventions (IMIs) and indicated IMIs as promising tools for stress prevention [29]. Thus, there is still a lack of systematical research focusing on the future user’s needs and demands. More evidence is needed to develop and implement tailored e-mental health approaches (electronic mental health) [30], [31] and investigate the impact and effectiveness of participatory research [32] within medical students and education [33].

This study wants to transmit results of patient-centered studies into medical education research to fill the gap of research focusing on medical students’ preferences within the development of tailored e-health approaches.

### 1.2. Objectives 

The objective of this study was to investigate the needs and demands for a tailored e-mental health app among medical students to foster stress management and promotion of personal skills within medical education. Previous research focusing on the acceptance and predictors of acceptance among medical students regarding the implementation and potential use of tailored e-mental health apps [34] laid the foundation of this secondary analysis and extension. A cluster analysis was conducted to investigate the presence of subgroups to get detailed insights regarding individual needs [35], [36], [37]. 

Key question addressed:

- What are the needs and demands of medical students regarding the design, content and integration of an e-mental health app within medical education in relation to possible cluster regarding mental health condition?

## 2. Methods

### 2.1. Study design and participants

A cross-sectional study was conducted at University of Duisburg-Essen, North-Rhine-Westphalia, Germany from November 2022 to July 2023. *N*=305 (100%) students attending the course of psychosomatic medicine within the 5^th^ clinical semester were invited to participate. All students were aged 18 or above. Of *N*=245 (80.3%) students participating, *N*=16 (6.5%) individuals could not be included in the final data analysis due to missing content. In total, *N*=229 (93.5%) students were included in the final data analysis.

### 2.2. Ethical considerations and reporting guideline

The survey was conducted according to the Declaration of Helsinki and approved by the Ethics Committee of the Medical Faculty of the University of Duisburg-Essen (21-10196-BO). The results were reported in compliance with the Strengthening the Reporting of Observational Studies in Epidemiology guidelines [38]. For participation no financial or any other compensation was offered. The survey was anonymous and voluntary. Written informed consent was obtained. 

### 2.3. Assessment instruments 

The cross-sectional study encompassed a paper-pencil assessment including established self-designed questionnaires to assess sociodemographic data, use of digital technologies, prior experience with and attitudes towards e-health apps, needs and demands regarding the development of a tailored e-mental health app. Mental health data such as symptoms of anxiety and depression were assessed using validated measures, PHQ-2 (Patient Health Questionnaire-2) and GAD-2 (Generalized Anxiety Disorder Scale-2).

#### 2.3.1. Sociodemographic data

Firstly, sociodemographic data regarding gender, age and marital status were assessed.

#### 2.3.2. Mental health data

The Patient Health Questionnaire-4 (PHQ-4), a validated and well-established four-item instrument was applied to measure the medical students’ anxiety and depression level [39]. Answers were given on a four-point Likert-Scale from 0 (“never”) to 3 (“nearly every day”). The PHQ-4 was divided into the two 2-item measures: PHQ-2 and GAD-2. The PHQ-2 screens for symptoms of depression and the GAD-2 screens for symptoms of general anxiety over a period of two weeks. Both set a cutoff score of ≥3 as an indicator for depression/anxiety [40], [41]. The distress among medical students was analyzed on a scale from 0 (“no distress”) to 10 (“extreme distress”) to cover the students’ distress in the past week. Additionally, self-generated questions contained information on prior contact to internal or external psychosocial support system.

#### 2.3.3. Needs and demands

Needs and demands were assessed by tailored items with different answer options (dichotomous, single- or multiple-choice, and Likert-Scales) and were designed and adapted by the research group based on prior studies [35], [36], [42], [43], [44]. The suitability of several formats were measured on a five-point Likert scale (1=“very unsuitable” to 5=“very suitable”). Main characteristics regarding the content and construction were analyzed. The students rated the importance of nine predetermined subjects on a five-point Likert scale (1=“unimportant” to 5=“very important”). Suitable contents and subjects were identified based on current literature and through expert interviews from the clinic of psychosomatic medicine and working-group of digital health at the LVR-University Hospital in Essen [42], [45], [46]. Further topics of interest or notifications could be provided using open-ended questions. 

#### 2.3.4. Prior experience and usage of digital media

Prior experience with online-based programs for health promotion and stress management, was assessed using adapted five-point Likert scales, along with information on the average usage of digital media. The Likert scales ranged from 1 (“do not agree”) to 5 (“totally agree”) and were developed based on current literature [42], [43].

#### 2.3.5. Statistical analysis 

Statistical analysis was performed using SPSS Statistic version 26 (IBM, New York, NY, USA), RStudio version 4.0.2 (RStudio PBC, Boston, MA, USA) and Microsoft Excel version 16.86 (Microsoft). Prior to any statistical test, relevant assumptions and prerequisites were tested. The level of significance was set at alpha=0.05. P-values were adjusted using Bonferroni correction for multiple tests. Descriptive statistics were performed. Cut-off scores for PHQ-2 and GAD-2 were computed. Mental health data (PHQ-2, GAD-2, distress score) was used to perform a cluster analysis to investigate hypothetical subgroups within the sample. *K*-medoids method, a conservative measure, was chosen because of its robustness against outliers. Hopkins’ *H* statistic was used to assess the clustering tendency of the data and the suitability for clustering. The parameter package was utilized to assess the optimal number of clusters [47]. A two-cluster analysis was conducted. The cohort was divided into a low- and high-burden cluster. The model’s overall performance was assessed using *R^2^* statistics and the classifiably was analyzed by linear discrimination analysis. Wilcoxon rank sum test was utilized to perform group comparisons of the clusters. Differences and similarities regarding the needs and demands of those affected by mental loads were analyzed descriptively. 

## 3. Results

### 3.1. Study population

*N*=229 medical students could be included in the final analysis with a mean age of 25.05 years (*SD*=2.83; *MIN*=20, *MAX*=37 years). The majority was female (68.6%, *n*=157). 39% were living in a relationship or married.

### 3.2. Mental health data

12.2% of the medical students reported elevated depression levels with PHQ-2 sum scores ≥3 and 22.3% elevated anxiety levels with GAD-2 sum scores ≥3. A detailed description regarding the mental health data is represented in table 1 (Tab. 1).

60.70% (*N*=139) acknowledged awareness of the support services for help-seeking to foster mental health available at the medical faculty, yet had not availed themselves of these resources. 13.97% (*N*=32) reported previous engagement with psychological consultation services. 9.61% (*N*=22) of respondents indicated a lack of prior knowledge regarding the availability of assistance at the medical faculty but expressed interest in utilizing such services. In contrast to that, 12.22% (*N*=28) of participants displayed disinterest in seeking help at the medical faculty and were unaware of the support options available. A minority, comprising 3.06% (*N*=7) of respondents, expressed a preference for seeking external assistance.

### 3.3. Prior experience and usage of digital media

Medical students reported spending an average of 1.42 hours per day (*SD*=1.82) utilizing digital media in occupational contexts outside of medical school, 3.81 hours per day (*SD*=2.06) for university-related purposes, and 3.23 hours per day (*SD*=1.76) for personal activities. Regarding e-health utilization and prior experiences with health-related information, 84.72% (*N*=194) of the students indicated frequent engagement with digital health solutions. Specifically, 40.35% (*N*=92) reported frequent use for physical activity and fitness, 26.64% (*N*=66) for self- and time-management, 8.73% (*N*=20) for stress-management, and 8.30% (*N*=19) for sleep tracking.

### 3.4. Needs and demands

Medical students rated various needs and demands regarding the implementation and design of an e-health app. 

#### 3.4.1. Availability and (un)suitable formats

The vast majority, 97.3% (*N*=222) of the students rated availability via smartphone (*M*=4.82, *SD*=0.52) and 91.2% (*N*=209) via tablet (*M*=4.55, *SD*=0.72) as most suitable. Video material (85.59%), download material (72.37%), audio material such as podcasts (70.31%) and contact to experts (70.31%) were considered suitable or very suitable for promotion of e-mental health within medical education by most of the students. Chatbot (36.40%) and games (31.44%) were seen as less suitable to unsuitable. A detailed overview of the response frequencies are represented in table 2 (Tab. 2). 

#### 3.4.2. Usage, usability and construction

Most of the students preferred using the app throughout their whole studies especially as required or on demand, e.g. in incriminating situations and stressful times. New content should be available one to three times per week (51.98%, *N*=118). Most students favored usability on demand and as required (79.91%, *N*=183) followed by a guided modular design (43.23%, *N*=99). Each session should be around 10 to 20 minutes (40.17%, *N*=92). A detailed overview is represented in table 3 (Tab. 3).

#### 3.4.3. Content and subjects

**Figure 1:**
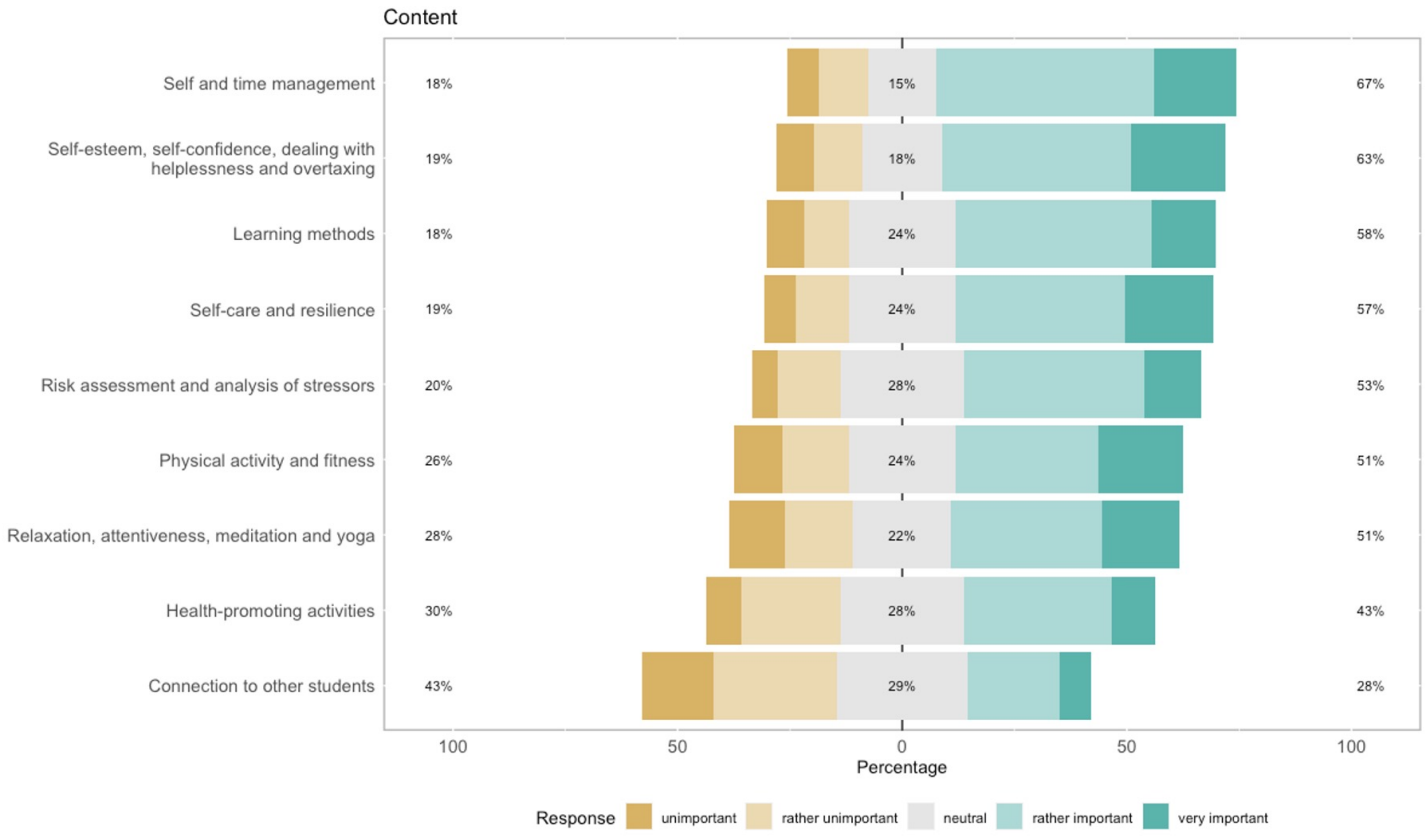
Content that should be addressed by an e-mental health application within medical education, N=229

The following issues were rated as most important and should be addressed: self-/time-management (66.81%) as well as self-esteem/-confidence and dealing with helplessness (63.16%). A detailed overview of responses is presented in figure 1 (Fig. 1).

#### 3.4.4. Cluster Analysis

To conduct a cluster analysis mental health data (i.e., PHQ-2, GAD-2, Distress score) were chosen for clustering. Using the Hopkins’ *H*-statistic, the data showed a clustering tendency (Hopkins’ *H*=0.41). The choice of two clusters was supported by 12 (41.38%) methods out of 29. The *k*-medoids method was used for performing a two-cluster analysis. The two clusters were interpreted as low burden cluster (*n*=111) and high burden cluster (*n*=118). The model performance was *R^2^*=0.342. The overall accuracy of classification was 93.01%. 

**Figure 2:**
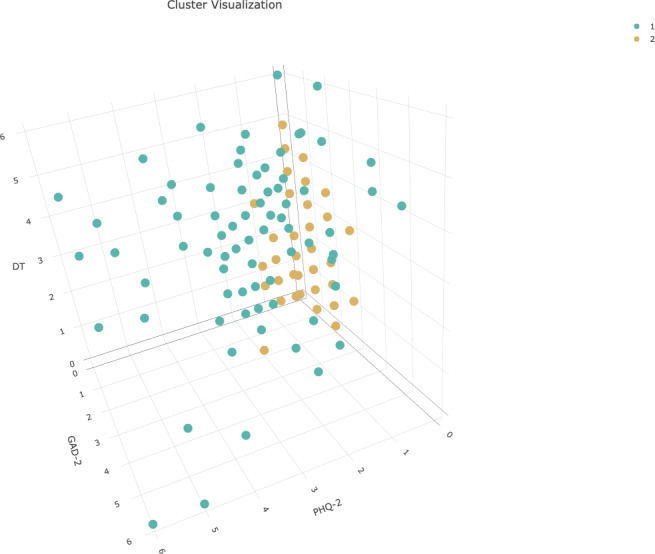
Cluster analysis (1=“high burden cluster”, 2=“low burden cluster”), N=229

The cluster analysis is visualized in figure 2 (Fig. 2). 

Significant differences between the low burden cluster and the high burden cluster were identified. Among the high burden cluster, the mean PHQ-2, GAD-2, and Distress sum scores were significantly higher than in the low burden cluster (p_adj_<.001). An overview of the group characteristics is represented in table 4 (Tab. 4).

#### 3.4.5. Comparison of the clusters

**Figure 3:**
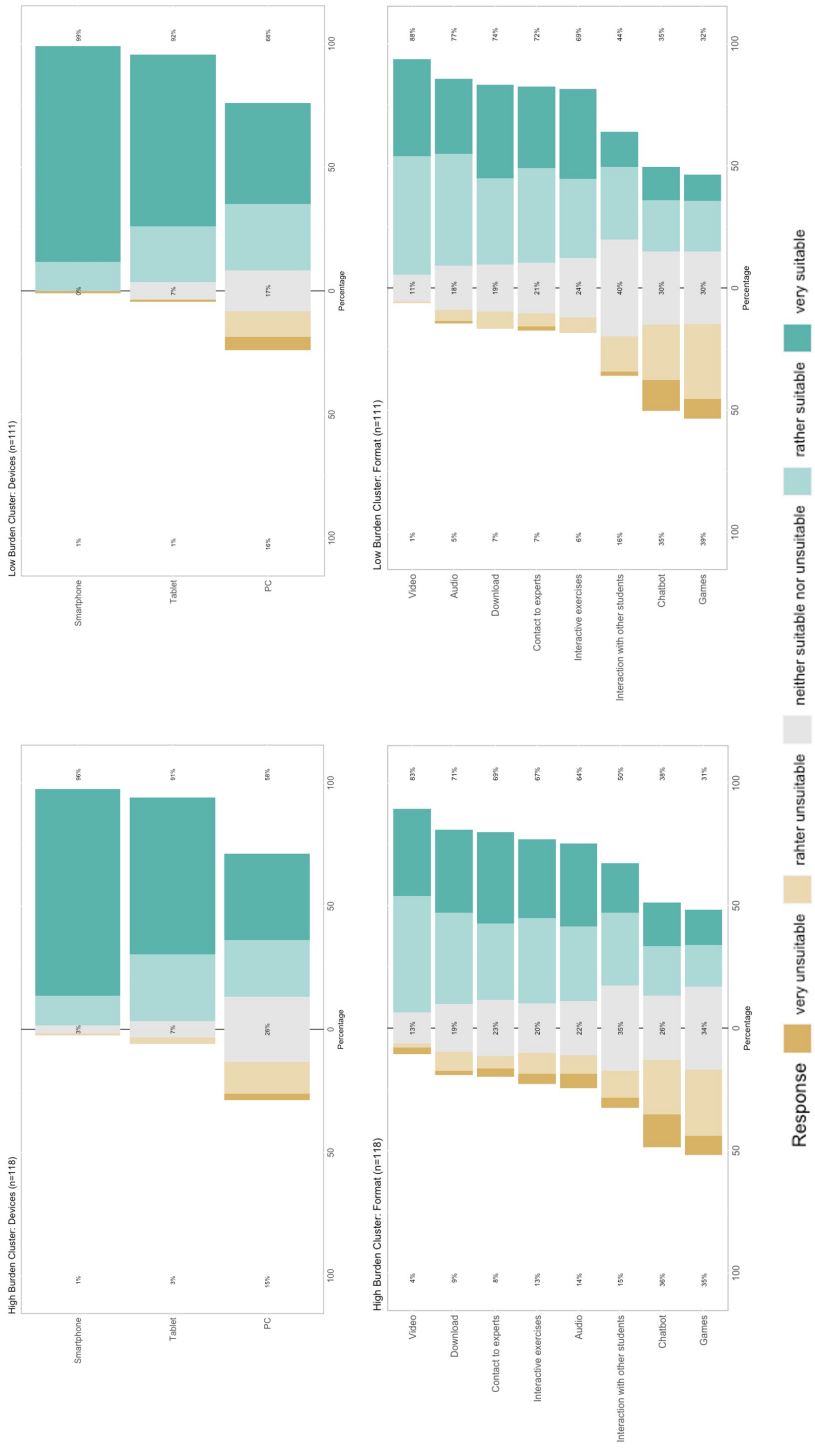
Response frequencies regarding the usefulness of devices and formats

**Figure 4:**
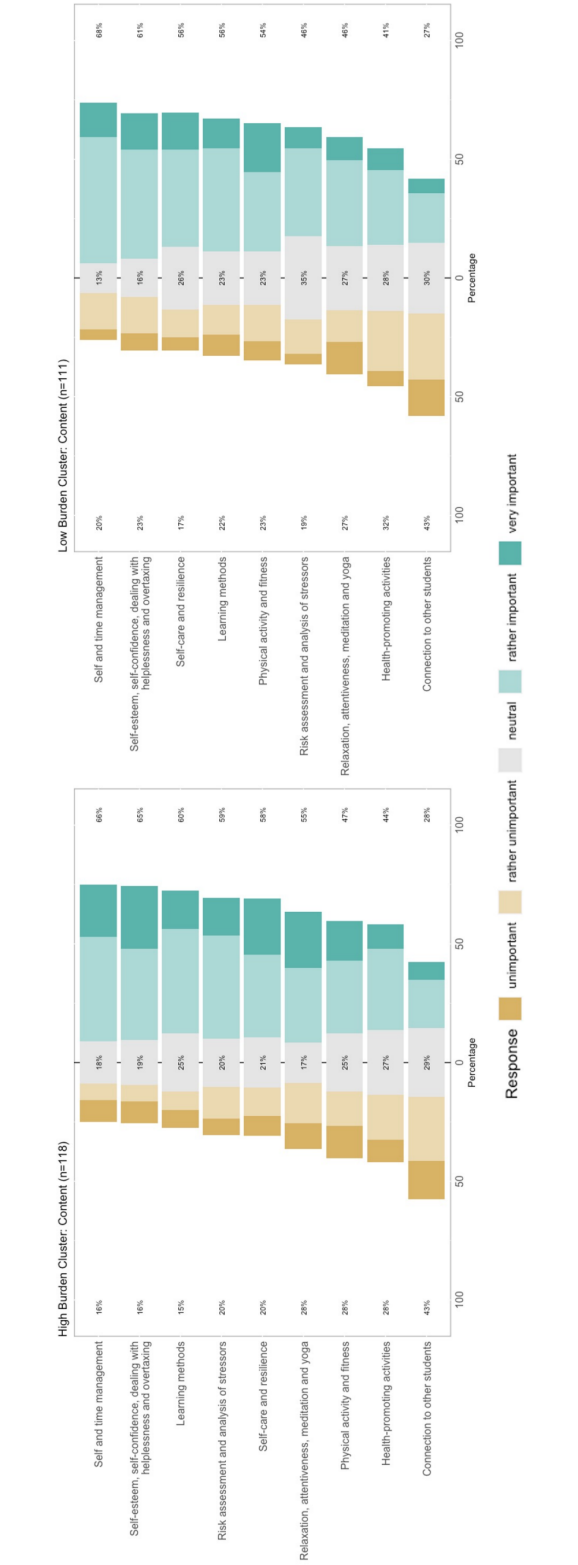
Response frequencies regarding the importance of different contents

Characteristics of the low and high burden cluster were compared descriptively with focus on preferred devices, formats and content. 50% of the students within the high burden cluster rated interaction with other students as rather suitable to very suitable compared to 44% of the students within the low burden cluster. Within the high burden cluster learning methods, risk assessment/analysis of stressors, self-care and resilience as well as relaxation/attentiveness/meditation/yoga were rated as most important. Students within the low burden group valued self-care and resilience, learning methods and physical activity and fitness as highly important. An overview of the preferred devices and formats is represented in figure 3 (Fig. 3). The importance of contents between both clusters is compared in figure 4 (Fig. 4).

Students within the high burden cluster preferred a duration of 1 to 10 minutes of each session, compared to the low burden cluster with a preference of 10 to 20 minutes. The favored usability and construction are shown in table 3 (Tab. 3).

## 4. Discussion

The primary analysis of the current data found that most of the students reported high acceptance of a tailored e-mental health app within medical education [34]. Accordingly, this study examined the needs and demands regarding content and design of an e-mental health app among medical students in consideration of possible clusters regarding mental health condition.

Elevated levels of anxiety and depression are well-known among medical students. Our findings underline the importance of tailored mental health support due to elevated anxiety, depression and distress levels. Even though, nearly 10% of the students reported to be interested in seeking help at our medical faculty, 15% of the students would not seek help at their own faculty. These findings align with previous research [48] and should be considered within the design of additional supportive programs. A low-threshold possibility can be a valuable tool to support and extend in-person consultation on-campus to dismantle barriers and optimize help-seeking among medical students.

Relating to the needs and demands, smartphones or tablets were mostly preferred devices aligning with previous patient-centered research [35], [49]. Within our study, most of the participants were comparatively young. Digital media was frequently used for university and personal activities. Due to this, it might be conceivable that the students assign a high value to apps on smartphones and tablets offering optimal accessibility and low-threshold opportunities [29]. Most of the students preferred using the app throughout their whole study, especially as required or on demand, e.g. in incriminating situations and stressful times. The students favored weekly updates with new content one to three times a week. One session should take 10 to 20 minutes. This might indicate the wish to implement the app continuously within their routines. A modular design and guidance was preferred by nearly four out of ten whereas most students wanted to use the content on demand. This wish for self-guided usage might underline a pursuit of autonomy indicating that the structure and consent should be presented succinctly. To optimize usability and suitability, the mostly preferred formats (video and audio materials) need to be developed accordingly. Additionally, contact to experts was considered suitable for promoting e-mental health and should be offered correspondingly. Various topics were rated as most important by the students and should be addressed such as self-/time-management, self-esteem/-confidence and dealing with helplessness, learning methodologies and self-care/resilience. Due to individual characteristics of the target groups different topics need to be considered and investigated when developing digital apps.

Explorative analysis revealed two clusters, a low and a high burden cluster. Within the high burden cluster distress, anxiety and depression scores were significantly higher compared to the low burden cluster. Formats such as video material, downloadable material, contact to experts, interactive exercises and audio material were valued as useful especially within the high burden cluster. Connectivity and networking with other students were highly valued by students with more distress or anxiety aligning with the study by Kappner et al in 2022 [50]. These findings underline the importance of social support and should be investigated further. Comparing both clusters regarding content and subjects, the high burden cluster valued learning methods, risk assessment/analysis of stressors, self-care and resilience as most important in contrast to students within the low burden cluster who esteemed self-care and resilience, learning methods as well as physical activity/fitness. To encounter individual characteristics qualitative interviews or assessments designed as educative workshops could be considered for future studies. A study by Dederichs et al described educative workshops as suitable tools within medical education research on participatory design approaches [51]. 

The findings of the study establish an introductory framework for further studies and the design of e-mental health apps within medical education. Future research should focus on the impact of user-centered approaches within the design of e-mental health interventions among target groups [27], [52], [53] to supplement and extend existing support, e.g. on-campus consultation, and focus on individual needs. Even though tailored e-mental health apps represent promising approaches, the imperative of the learning environment and adaptation of the requirements within medical education should be focused.

### 4.1. Limitations

When interpreting our analysis, the following limitations should be considered. All data was collected within medical students attending psychosomatic seminars during the 5^th^ clinical semester, selection bias needs to be considered. The needs and demands at other medical faculties as well as within other semesters should be investigated further. This data based on self-reporting. The development of the survey based on the expertise of our working unit and from patient-centered studies. The needs and characteristics of male and divers/non-binary are highly essential and should be addressed within future studies. Despite the limitations and biases of our study the realization as paper-pencil-survey needs to be noted. Despite biases may limit generalizability and representativeness, our study provides a practical and easy-to-use approach to evaluate the needs and characteristics of medical students within the development and designing process of a tailored e-mental health app.

### 4.2. Implications

Medical students’ (in general: the future user’s) preferences should be linked to actual usage and adherence to mobile apps in future research and need to be explored further. On basis of our finding, a tailored e-mental health app for medical students can be developed and implemented. Additionally, designers, and other stakeholders should be directly involved within the designing process. More research is needed to investigate the effectiveness of tailored design approaches.

## 5. Conclusions

By developing e-mental health apps through a more user-centered design approach, highly beneficial approaches can be designed with optimized usability to support medical students’ mental health. This information may give important and useful advice to create a tailored app and should be considered within future research as well as directly within the designing process to optimize usability, adherence and help-seeking behavior. Our results will be used to design, develop, implement and evaluate an e-mental health app within medical education. 

## Abbreviations

- e-mental health: electronic mental health
- GAD-2: Generalized Anxiety Disorder Scale-2
- PHQ-2: Patient Health Questionnaire-2
- PHQ-4: Patient Health Questionnaire-4

## Notes

### Data availability

The datasets used and/or analyzed during the current study are available on reasonable request. Please contact the corresponding author Dr. med. Anita Robitzsch (anita.robitzsch@lvr.de). 

### Funding

The study was conducted without external funding. We thank the Open Access Fund of the University of Duisburg-Essen for their support during the publication of our article.

### Authors’ contributions

AR, MT, and AB initiated and conceptualized the study. Project administration was conducted by AR, AB, and SB. CG and SK performed the statistical analyses and interpretation of the data. CG wrote the original draft of the manuscript. Data acquisition was performed by AR, AB, and CG. AR, MT, AB, and SB supervised the project and contributed to the study design and data collection. SB, DD, CJ and ND gave important input regarding the critical revision of the manuscript. All authors contributed to the further writing of the manuscript and approved its final version. 

### Authors’ ORCIDs

- Catharina Grüneberg: [0009-0002-0918-5905]
- Alexander Bäuerle: [0000-0003-1488-8592]
- Sophia Karunakaran: [0009-0000-1202-2950]
- Dogus Darici: [0000-0002-2375-8792]
- Nora Dörrie: [0000-0002-9760-9167]
- Sven Benson: [0000-0002-4487-4258]
- Martin Teufel: [0000-0003-2120-1840]
- Anita Robitzsch: [0009-0005-2941-1123]

## Acknowledgements

We thank Prof. Dr. med. Joachim Fandrey, for his support and input regarding the critical revision of the manuscript. We thank Lisa Jahre and Anna-Lena Frewer for their support during the statistical analysis. 

We acknowledge support by the Open Access Publication Fund of the University of Duisburg-Essen.

## Competing interests

The authors declare that they have no competing interests. 

## Figures and Tables

**Table 1:**
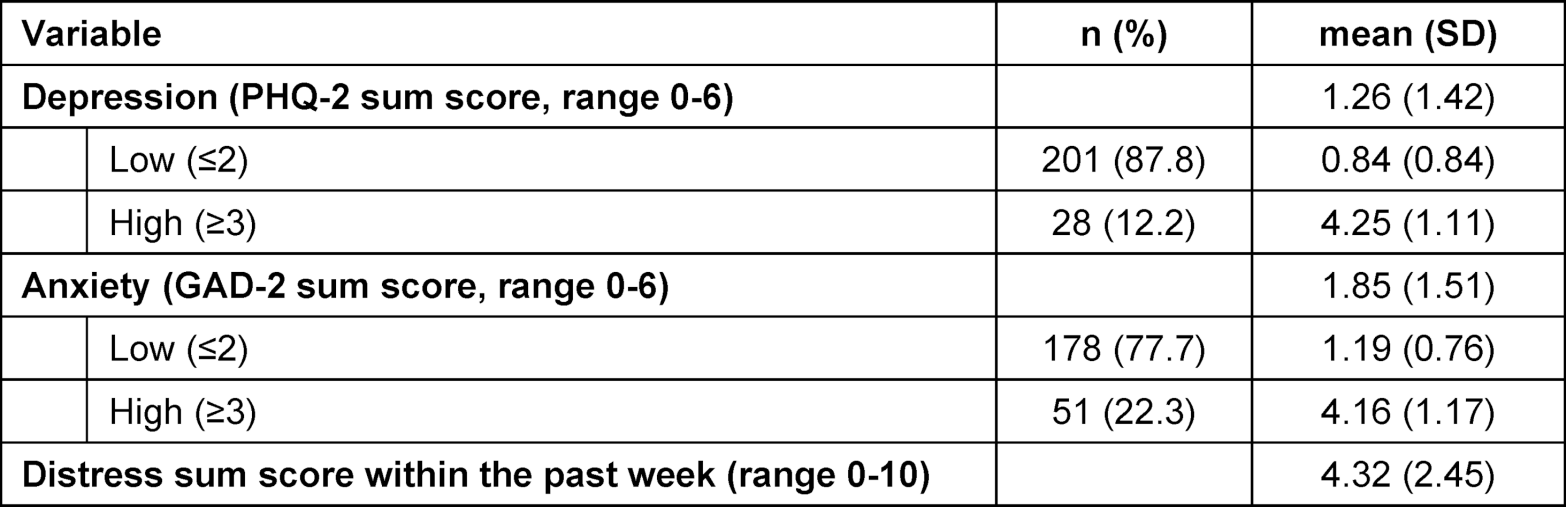
Mental health data of participants (N=229)

**Table 2:**
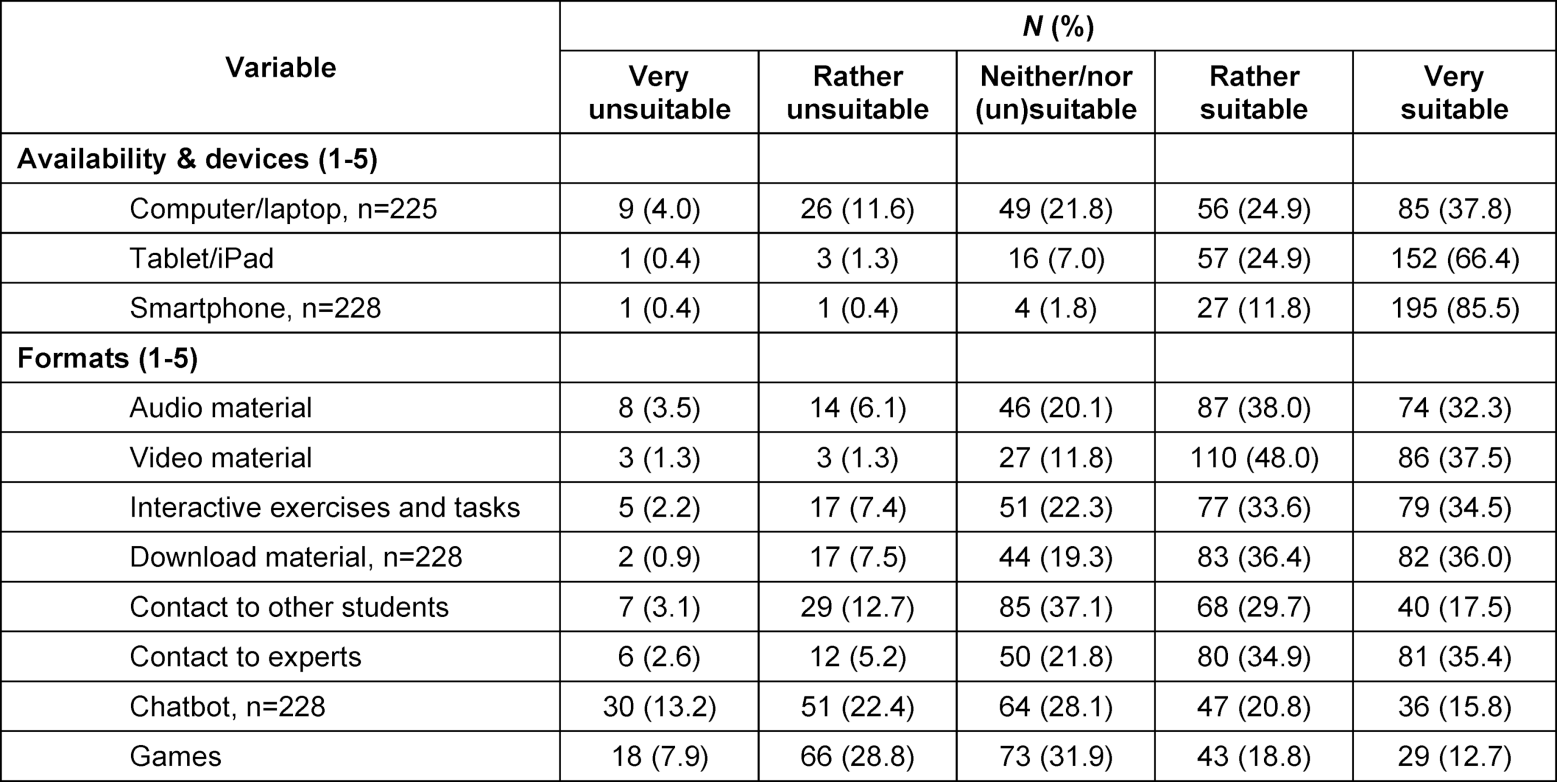
Response frequencies (total number and percentage) concerning availability and formats

**Table 3:**
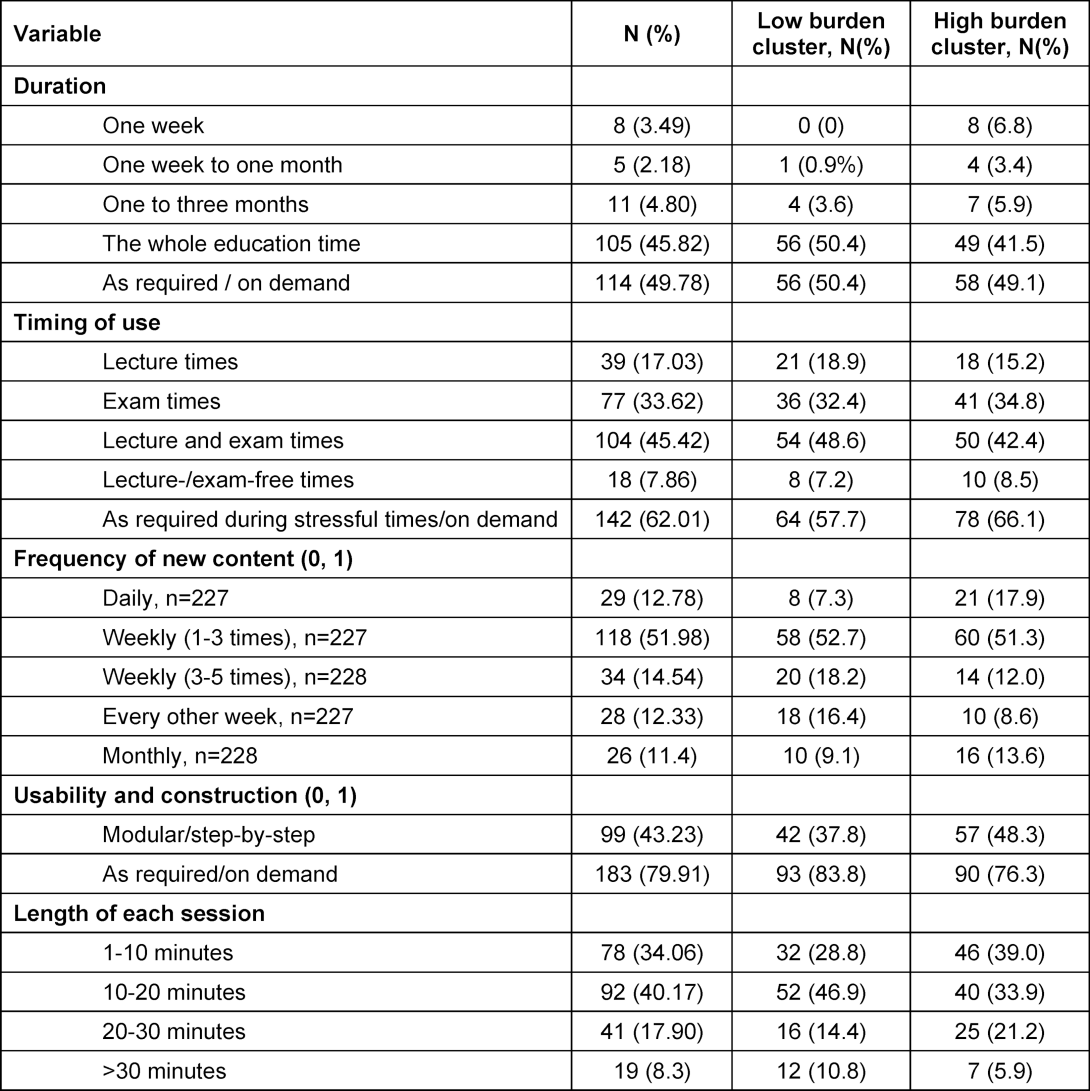
Number of responses (total number and percentage) concerning timing of use, availability and duration, frequency of new content and length of sessions (n=229)

**Table 4:**
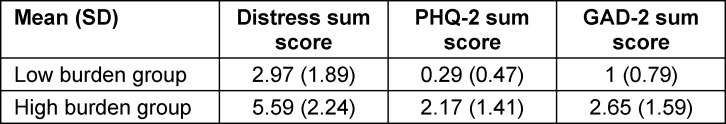
GAD-2, PHQ-2 and distress score by cluster. Mean (SD)
